# Effect of Narrow Band Ultraviolet B Therapy versus Methotrexate on Serum Levels of Interleukin-17 and Interleukin-23 in Egyptian Patients with Severe Psoriasis

**DOI:** 10.1155/2013/618269

**Published:** 2013-12-31

**Authors:** Tarek Mahmoud Elghandour, Sahar El Sayed Youssef, Dalia Gamal Aly, Mohamed Said Abd Elhameed, Mehrevan Mostafa Abdel Moneim

**Affiliations:** ^1^Department of Dermatology, Venereology, and Andrology, Ain Shams University, Abassya Square, Cairo 11566, Egypt; ^2^Department of Dermatology and Venereology, National Research Center, El-Bhoos Street, Dokki, Giza 12622, Egypt; ^3^Department of Medical Biochemistry, National Research Center, El-Bhoos Street, Dokki, Giza 12622, Egypt

## Abstract

*Background*. There is raised interest in the involvement of interleukin-(IL-)23/T-helper 17 cells (Th17) axis in the pathogenesis of psoriasis. *Objectives*. To compare the effect of narrow band ultraviolet B (NB-UVB) and methotrexate (MTX) therapy on serum levels of IL-17 and IL-23 in psoriatic patients. *Methods*. Thirty patients with severe plaque psoriasis were included: 15 patients received NB-UVB three times weekly (group I) and 15 patients received MTX 0.3 mg/kg per week (group II), both for 8 weeks. Before and after treatment, serum levels of IL-17 and IL-23 were investigated by ELISA technique and psoriasis area and severity index (PASI) was calculated. *Results*. After treatment, all patients showed a reduction in their PASI score, IL-17 and IL-23 serum levels with a nonsignificant difference between both therapeutic modalities (*P* value >0.05). A positive correlation was detected between the percent of reduction of IL-17, IL-23 and the percent of reduction of PASI score for patients receiving both treatments. No correlation was found between the percent of reduction of IL-17, IL-23 and duration of disease or age of all patients in this study. *Conclusion*. Interleukin-17 and IL-23 serum level may serve as a potential biomarker for predicting the prognosis and therapeutic response of NB-UVB or MTX in treating psoriasis.

## 1. Introduction

Psoriasis is a cutaneous disorder characterized by epidermal hyperproliferation and inflammation with a marked infiltration of T cells, neutrophils, and macrophages. Psoriatic inflammation was once considered to be mediated by T-helper 1 (Th1) cells; however, recent studies increasingly indicate that immune responses by newly characterized Th17 cells are also involved in the pathogenesis of psoriasis [1, 2]. The findings of several reports of elevated levels of interleukin- (IL-) 23 and Th17 related cytokines in cutaneous lesions and in the serum of psoriatic patients, as well as the association of IL-23 receptor gene (IL-23R) variants with psoriasis, provide the basis for a rising interest in the IL-23/Th17 axis in psoriasis [2].

T-helper 17 cells are inflammatory CD4+ T cells that do not produce interferon- (IFN-) *γ* or interleukin- (IL-) 4; instead, they produce IL-6, IL-17, IL-21, IL-22, and tumor necrosis factor (TNF)-*α* [3]. Interleukin-17 family, for which the Th17 cells lineage is named, consists of six cytokines (IL-17A-F). IL-17A, IL-17B, IL-17C, and IL17F have proinflammatory properties, can induce cytokines such as TNF and IL-1, and promote neutrophil migration. Interleukin-17 also expresses the IL-23R. Although Th17 cells are the major source of IL-17, it can be produced by a wide range of cell types, including CD8+ cells, neutrophils, B cells, natural killer T cells, gamma delta T cells, and LTi cells [4].

Interleukin-23, a member of IL-12 family, is a heterodimeric cytokine formed by a p40 chain, which is shared with IL-12. Interleukin-23 is secreted by dendritic cells, macrophages, other antigen-presenting cells, and keratinocytes and plays a pivotal role in the survival and proliferation of Th17 cells after priming with transforming growth factor- (TGF-) *β* and IL-6 [5].

The successful use of most conventional antipsoriasis treatment was generally based on serendipity, and the exact mode of action was often poorly understood. In this new era of IL23/Th17 in the pathogenesis of psoriasis, the effect of the conventional therapy deserves further investigation [4].

Narrow-band ultraviolet (NB-UVB) therapy is known to reverse several pathologic alterations in psoriasis as it decreases the number of epidermal T lymphocytes and dendritic cells during therapy [6]. In addition to its known role in suppressing IFN-*γ* production, NB-UVB radiation therapy was found to target the IL-17 pathway [7].

Methotrexate (MTX), an effective therapy for patients with psoriasis, was not able to cause significant reduction in the blood levels of IL-23, IL-17, IL-22, and Th17 cells in patients with rheumatoid arthritis [8, 9]. However, in patients with psoriasis, Meephansan et al., 2011 [10], showed that MTX significantly reduced serum level of IL-22 and there was a positive correlation between IL-22 levels and psoriasis area and severity index (PASI) score. To our knowledge, there are no studies in the literature, particularly in Egyptian psoriatic patients, comparing the effects of NB-UVB and MTX, on the serum levels of IL-17 and IL-23. Therefore, the aim of this work was to study the influence of these two lines of therapy on the serum levels of IL-17 and IL-23 in patients with severe plaque psoriasis.

## 2. Materials and Methods

### 2.1. Patients

This study included thirty patients with severe plaque psoriasis without clinical joint involvement, who were required to have a baseline PASI score of ≥20. All the patients were comparable in terms of previous treatments and phase of psoriasis activity. They were divided into 2 groups: 15 patients were treated with NB-UVB (group I) and 15 patients were given MTX (group II), both for eight weeks. Patients received no other psoriasis treatments during the course of the study. They were randomly assigned to one of the two groups. We excluded all patients receiving any systemic treatment suppressing the immune system, such as systemic steroids, MTX or other immune suppressive drugs for the last 6 weeks, and topical medications for the last 2 weeks, prior to sample collection, patients having any systemic or dermatological disease affecting the immune system, patients under 18 years, pregnant and lactating females, and patients having liver or renal disease.

All patients were randomly selected from the Dermatology Outpatient Clinics of the National Research Center and Ain Shams University Hospitals. An informed consent was taken from all patients before participating in this work. The study was approved by the Ethical Committee of the National Research Centre, Giza, Egypt.

### 2.2. Methods

All patients were subjected to detailed history taking and general and dermatological clinical examination. At the baseline and one week after the end of the treatment (at week 9), blood samples were taken to evaluate IL-17 and IL-23 levels and a blind clinical assessment by calculating PASI score was made. The PASI score evaluates the severity of psoriasis in relation to three parameters: E, erythema; I, infiltration; and D, desquamation [11]. It was then calculated according to the formula in the published work.

### 2.3. Assessment of Serum Levels of Interleukin-17 and Interleukin-23

Interleukin-17 and Interleukin-23 assessment were performed for all patients after the collection of samples had been completed. Five mL venous blood samples were withdrawn from each patient on the initial visit before receiving treatment and another sample was withdrawn nine weeks after receiving the treatments (with either NB-UVB or MTX). Samples were then centrifuged, and the separated sera were kept frozen at −20°C until analysis. IL-17 and IL-23 assay were done using the WKEA MED Supplies Enzyme-Linked Immunosorbent Assay (ELISA) kit, New York, USA. The experimental procedures were performed according to the manufacturers' instructions in a blinded fashion on coded samples by an investigator who was not informed of the patients' clinical status.

### 2.4. Treatment Protocol

#### 2.4.1. Narrow-Band Ultraviolet B

Fifteen patients (group I) were treated with NB-UVB. The initial radiation dose was determined according to the patient's skin type as follows: those with skin types I and II were initiated with 0.3 mJ/cm^2^, and those with skin types III or IV were treated with 0.5 mJ/cm^2^. Dose increment was done every session according to the degree of erythema as follows: if no erythema, dose increment was 20%, minimum erythema, dose increment was 10%, if intense erythema or erythema and edema or erythema, edema and blisters, no dose increment was done [12].

Sessions were given three times weekly with 48 hours apart for eight weeks. The machine used was UV-100L Waldman (Germany) lighting and was equipped with UVB lamps (TL01 lamp) which have physical irradiance values of 7–10 mW/cm^2^ and biological effective (erythematous) irradiance of 0.4–0.6 mW/cm^2^.

#### 2.4.2. Methotrexate Administration

Fifteen patients (group II) were treated with MTX (0.3 mg/kg/week) for eight weeks. Complete blood count, liver function tests, and renal function tests were done for every patient before the first dose of MTX and every four weeks thereafter. Methotrexate was given by oral route once weekly. The dose was divided into three parts given 12 hours apart over 24 hours. Folic acid (5 mg/day) was given with the MTX course every day except for the days of the MTX dose.

#### 2.4.3. Statistical Analysis

Data was analyzed using the statistical program for social science (SPSS) 15.0.1 for windows; SPSS Inc., Chicago, IL, 2001. Mean and standard deviation were used to describe continuous data and number and percentage of categorical data. Paired samples *t*-test was used to assess the statistical significance of the difference between two means of one quantitative variable measured twice for the same study group, while Pearson's correlations was used to assess the strength of association between two quantitative variables. *P* values < 0.05 were considered statistically significant.

## 3. Results

Of the 30 psoriatic patients enrolled in the study, 18 were males (60%) (6 (40%) in group I and 12 (80%) in group II) and 12 were females (40%) (9 (60%) in group I and 3 (20%) in group II). Their ages ranged from 19 to 70 years, and mean ages in groups I and II were 39.2 ± 18.1 years and 45.4 ± 12.2 years, respectively. The duration of the disease was from 1.5 to 20 years in group I and 1 to 30 years in group II with a mean ± SD of 8.0 ± 5.5 and 14.2 ± 9.7 years, respectively.

### 3.1. Comparison between Narrow-Band Ultraviolet B (Group I) and Methotrexate (Group II) as regards the PASI Score

At baseline, both groups I and II showed no statistical difference regarding PASI score (mean ± SD 21.1 ± 0.6 and 24.7 ± 0.9, resp.). However, a highly significant decrease in the PASI score of both groups was noted after the eight week lasting therapy, as patients in group I attained a 53% reduction in their PASI score with a mean ± SD of 15.4 ± 1.2 and patients in group II attained a 60% reduction in their PASI score with a mean ± SD of 16.8 ± 1.1 (*P* value < 0.05) for both groups. However, a nonsignificant difference between both groups was noted (*P* value  >0.05).

### 3.2. Comparison between Serum Levels of Interleukin-17 and Interleukin-23 before and after Treatment with Each of Narrow-Band UVB and Methotrexate

Narrow-band UVB achieved a highly significant reduction in both IL-17 and IL-23 levels as compared to baseline levels (mean ± SD before treatment 2.0 ± 1.5 ng/L and 499.1 ± 207.1 ng/L versus 1.32 ± 1.2 ng/L and 281.7 ± 214.1 ng/L post treatment, resp., *P* value = 0.041 and <0.001 resp.) (Table 1). Furthermore, MTX also achieved a highly significant reduction in both IL-17 and IL-23 levels as compared to baseline levels (mean ± SD before treatment 2.2 ± 0.6 and 440.9 ± 193.8 versus 1.1 ± 0.5 and 276.0 ± 179.3 after treatment, resp., *P* value < 0.001 and 0.022, resp.) (Table 1).

On comparing both groups as regards the reduction of PASI score, IL-17, and IL-23, a nonsignificant difference was noted (*P* value  >0.05) (Table 2). Moreover, there was also a nonsignificant difference between the percent of reduction of either IL-17 or IL-23 and the age or gender among the studied groups (*P* value > 0.05).

There was a positive correlation between the percent of reduction of both IL-17 and IL-23 after treatment and the reduction in PASI score among the studied groups (*r* = 0.76 and 0.89 in group I and II for IL-17, resp., versus *r* = −0.91 and −0.64 in groups I and II for IL-23, resp., *P* value = 0.03 and 0.01, resp.). However, there was no correlation between the percent of reduction of either IL-17 or IL-23 after treatment and the duration of disease (*r* = −0.07 for IL-17 versus −0.38 for IL-23) or age (*r* = 0.18 for IL-17 versus 0.12 for IL-23) in all patients included in this study (*P* value > 0.05 for both).

## 4. Discussion 

The clinical benefit from conventional and biologic systemic therapies targeting psoriasis was found to correlate with IL-17 and IL-23 downregulation in psoriatic patients [7, 13]. In the current work, our data confirmed that both NB-UVB and MTX were able to induce a significant reduction in the PASI index after 8 weeks of therapy, with MTX being more effective than NB-UVB as the reduction in PASI score was 60% after MTX versus 53% after NB-UVB. However, it did not reach any statistical significance. Nevertheless, the most important aim of our study has been the comparison between NB-UVB and MTX effects on the serum levels of IL-17 and IL-23. Our data demonstrated a reduction of IL-17 and IL-23 serum levels after therapy with both NB-UVB and MTX in psoriasis patients with a nonsignificant difference between both therapeutic modalities as well.

Our findings regarding NB-UVB are in accordance with several studies in the literature. Rácz et al., 2011 [14], noted a downmodulation of Th17 and a decrease in IL-17 after NB-UVB treatment. Similarly, Coimbra et al., 2010 [15] reported that TNF-*α*, IL-23, IL-22, and IL-17 decreased significantly after both NB-UVB and PUVA treatment with a significant reduction in PASI score and suggested that the reduction in the IL-23/Th17 axis might be important in the pathogenic mechanisms of psoriasis. Moreover, Johnson-Huang and coworkers, 2010 [7], also demonstrated that NB-UVB suppressed multiple parameters of the IL-23/IL-17 pathway in psoriatic lesions.

We still do not fully understand the mechanism of action of this remarkably effective therapy. At the cellular level, NB-UVB therapy has many immunosuppressive effects. It leads to a reduction of T cells by inducing apoptosis [16, 17]. Keratinocyte apoptosis also occurs as a result of in vitro NB-UVB [17]. In addition, some studies have found a decrease in the numbers of Langerhans cells after therapy [18], although others have not found a significant decrease [6, 16]. Nevertheless, it is clear that NB-UVB impairs in vitro antigen presentation by both murine dendritic cells and human Langerhans cells, rendering them tolerogenic rather than stimulatory. Thus, NB-UVB can suppress a broad range of immune cells [7].

Lately, there have been efforts to understand the effect of NB-UVB on inflammatory cytokine production. Narrow-band UVB specifically targets IFN-*γ*-producing Th1 cells as well as upstream cytokines, IL-12 and IL-23 [19]. It also has suppressive effects on additional inflammatory mediators, including IL-18, IL-8, IL-1b, and IL-6. Thus, NB-UVB can suppress inflammatory cytokines as well [7].

Some studies in the literature have also suggested that the clearing of the inflammatory cells is specifically related to how far the UV radiation can penetrate [20]. At the cellular level, sufficient penetration of NB-UVB into the upper dermis results in the clearing of myeloid dendritic cells and T cells, which seems necessary for reduction of inflammatory cytokine production. Furthermore, the dermal T cells that remain after NB-UVB produce less inflammatory cytokines, such as IFN-*γ* [21]. We therefore recommend the assessment of IL-17 and IL-23 production by lesional T cells in future studies.

However, it is still unclear whether suppressing the IL-23/IL-17 axis, as demonstrated in our work, is simply because of the depletion of immune cells by NB-UVB or whether the therapy itself can specifically inhibit cytokine production. Studies using flow cytometry have shown that UVB can directly inhibit cytokine production by T cells [22]. With this respect, we also report that NB-UVB was able to suppress T-cell production of IL-17 and IL-23 in the current work.

As far as we know, our study was the first to assess the effect of MTX on serum levels of IL-17 and IL-23. Methotrexate is still used as a cost-effective treatment for moderate to severe psoriasis. It is known to inhibit DNA synthesis by competing as a substrate for dihydrofolate reductase, resulting in an antiproliferative effect. However, this does not fully explain the effectiveness of MTX in psoriasis. In vitro studies have suggested that MTX has an anti-inflammatory effects through induction of immune cell apoptosis and inhibition of T-cell activation [10].

The mechanism by which MTX reduces IL-17 and IL-23 is speculative at this point. Both interleukins are produced by several cell types including Th-17, CD8+ T, natural killer T cells and dendritic cells [23]. The antiproliferative effect of MTX was originally considered to be the fundamental therapeutic mechanism in psoriasis. In this regard, reduced levels of these cytokines could be the consequence of an antiproliferative effect of MTX on dermal dendritic cells and varieties of T-cells. However, studies have not found reduced proliferation of these cell types to be a consistent finding in psoriasis. Both in vivo and in vitro studies reported that MTX could reduce T-cell numbers in psoriatic skin and in the circulation but had no effect on interferon-*γ* and IL-4 production. Methotrexate can suppress inflammation in psoriasis by reducing the numbers of T cells and monocytes in the skin along with reduced expression of adhesion molecules [22, 24].

Methotrexate also acts on immune cells as well as keratinocytes, and both these cell types play important roles in the pathogenesis of psoriasis, serving as potent sources of proinflammatory cytokines, chemokines, and growth factors. Thus, it is possible that reduced levels of IL-17 and 23 may result from the anti-inflammatory effects of MTX [10]. The present study presents evidence for a novel therapeutic mechanism of methotrexate in psoriasis via reduction of serum IL-17 and IL-23. However, additional clinical studies would be of value to confirm this finding. With methotrexate being as effective as NB-UVB, the risks and benefits should be weighed before using MTX as a first line of treatment in severe psoriasis.

In the current work, in addition to the significant reduction in the PASI score and both IL-17 and IL-23 after receiving either MTX or NB-UVB, we found a positive correlation between the percent of reduction of IL-17 or IL-23 levels and the percent of reduction in the PASI score for both modalities of treatment. Arican et al., 2005 [25], stated that IL-17 serum levels had a significant correlation with PASI score. Similarly, Takahashi et al, 2010 [26], found that serum levels of TNF-*α*, IFN-*γ*, IL2, IL-6, IL-7, IL-8, IL-12, IL-17, IL-18, and VEGF were positively correlated with PASI in Japanese patients with psoriasis. Further studies on a larger scale of patients are, however, required to confirm or refute our findings.

To sum up, serum levels of IL-17 and IL-23 decreased after both NB-UVB and MTX therapy confirming the importance of this axis in the pathogenesis of psoriasis and providing evidence, in particular, for a novel therapeutic mechanism of MTX in the disease. Measuring their serum levels using ELISA technique is an easy, rapid, and feasible test for determination of disease activity and therapy evaluation of psoriatic patients. Additional studies would be of value to confirm our finding.

## Figures and Tables

**Table 1:** Comparison between serum levels of interleukin-17 and Interleukin-23, before and after narrow band UVB among groups I and II patients.

Interleukins	Group I (number = 15)			Group II (number = 15)		
	Before treatment	After treatment	*P* value	Before treatment	After treatment	*P* value
IL-17 ng/L	2.0 ± 1.5	1.3 ± 1.2	0.041	2.2 ± 0.6	1.1 ± 0.5	<0.001^†^
IL-23 ng/L	499.1 ± 207.1	281.7 ± 214.1	<0.001^†^	440.9 ± 193.8	276.0 ± 179.3	0.022^†^

^†^A significant difference by paired samples t-test.

**Table 2:** Comparing patients in groups I and II as regards PASI score, interleukin-17, and interleukin-18 before and after treatment with both narrow band UVB and methotrexate.

Patient group (Number = 15)	Mean	Standard deviation	*t*	*P* value	Significance
PASI (before treatment)					
Group I	21.1	0.6	−1.43	0.1	Nonsignificant
Group II	24.7	0.9			
PASI (after treatment)					
Group I	15.4	1.2	−0.12	0.9	Nonsignificant
Group II	16.8	1.1			
IL-17 (ng/L) before treatment					
Group I	2.0	1.5	−0.36	0.7	Nonsignificant
Group II	2.2	0.7			
IL-17 (ng/L) after treatment					
Group I	1.3	1.2	0.40	0.6	Nonsignificant
Group II	1.1	0.5			
IL-23 (ng/L) before treatment					
Group I	499.1	207.1	0.79	0.4	Nonsignificant
Group II	440.9	193.8			
IL-23 (ng/L) after treatment					
Group I	281.7	214.1	0.08	0.9	Nonsignificant
Group II	276.0	179.3			

The independent samples *t*-test.
